# The challenge of care coordination by midwives during the COVID-19 pandemic: a national descriptive survey

**DOI:** 10.1186/s12884-022-04772-2

**Published:** 2022-05-25

**Authors:** Laurent Gaucher, Corinne Dupont, Sylvain Gautier, Sophie Baumann, Anne Rousseau

**Affiliations:** 1 Geneva School of Health Sciences, HES-SO University of Applied Sciences and Arts, Geneva, Western Switzerland Switzerland; 2 Research On Healthcare Performance (RESHAPE), Université Claude Bernard Lyon 1, INSERM U1290, 69008 Lyon, France; 3 Hospices Civils de Lyon, Hôpital Femme Mère-Enfant, 69500 Bron, France; 4 Réseau Périnatal AURORE, 69004 Lyon, France; 5 Paris-Saclay University, Inserm U1018, Versailles Saint Quentin University, 78180 Montigny-le-Bretonneux, France; 6 Département Hospitalier d’épidémiologie Et de Santé Publique Assistance Publique des Hôpitaux de Paris, Hôpital Raymond Poincaré, 92380 Garches, France; 7 Midwifery Department, Paris-Saclay University, Versailles Saint Quentin University, 78180 Montigny-le-Bretonneux, France; 8 Department of Obstetrics and Gynecology, Poissy-Saint Germain Hospital, 78300 Poissy, France; 9 Paris-Saclay University, CESP, Epidémiologie Clinique, Versailles Saint Quentin University, 78180 Montigny-le-Bretonneux, France

**Keywords:** Midwife practice, Health service research, Survey study

## Abstract

**Background:**

As part of a decades-long process of restructuring primary care, independent (also known as community) healthcare workers are being encouraged to work in groups to facilitate their coordination and continuity of care in France. French independent midwives perform about half of the early prenatal interviews that identify mothers' needs during pregnancy and then refer them to the appropriate resources. The French government, however, structured the COVID-19 pandemic response around public health institutions and did not directly mobilise these community healthcare workers during the lockdown phase. These responses have raised questions about their role within the healthcare system in crises. This survey’s main objectives were to estimate the proportion of independent midwives who experienced new difficulties in referring women to healthcare facilities or other caregivers and in collaborating with hospitals during the first stage of this pandemic. The secondary objective was to estimate the proportion, according to their mode of practice, of independent midwives who considered that all the women under their care had risked harm due to failed or delayed referral to care.

**Methods:**

We conducted an online national survey addressed to independent midwives in France from 29 April to 15 May 2020, around the end of the first lockdown (17 March–11 May, 2020).

**Results:**

Of the 5264 registered independent midwives in France, 1491 (28.3%) responded; 64.7% reported new or greater problems during the pandemic in referring women to health facilities or care-providers, social workers in particular, and 71.0% reported new difficulties collaborating with hospitals. Nearly half (46.2%) the respondents considered that all the women in their care had experienced, to varying degrees, a lack of or delay in care that could have affected their health. This proportion did not differ according to the midwives’ form of practice: solo practice, group practice with other midwives only, or group practice with at least two types of healthcare professionals.

**Conclusions:**

The pandemic has degraded the quality of pregnant women’s care in France and challenged the French model of care, which is highly compartmentalised between an almost exclusively independent primary care (community) sector and a predominantly salaried secondary care (hospital) sector.

**Supplementary Information:**

The online version contains supplementary material available at 10.1186/s12884-022-04772-2.

## Background

Since 1978, the World Health Organization (WHO) has considered that community and individual self-reliance are the most reliable route to widespread, equitable, and sustained improvements in health [1]. Quality communication between primary and secondary care is essential to providing quality care [2]. A restructuring of the primary care sector began in the 1990s [3]. By 2018, the French government had declared that “isolated practice — i.e. that of a health professional alone in his or her practice — must become the exception by 2022” and was encouraging family physicians as well as independent midwives and other independent healthcare practitioners to practice in groups [4]. French midwives are responsible for only a small proportion of antenatal care, but this figure is increasing: in 2010 midwives monitored 11.6% of pregnancies, while that number rose to 23.3% in 2016, 14.8% by hospital midwives and 8.5% by independent community midwives [5]. Independent midwives also performed 47.2% of Early Prenatal Interviews (EPI) in 2016 [5]. This EPI is an official part of early prenatal care in France, intended to elicit and discuss the mothers’ expectations and needs and to provide them with the information or resources necessary to deal with their concerns [6].

On 31 December, 2019, the WHO issued an alert about several cases of pneumonia in the city of Wuhan in China [7, 8]. By 11 March, 2020, cases had occurred in every European country; the WHO reported a total of 17,413 cases that day, and its Director General classified COVID-19 as a “global pandemic” [9]. The following day, the European Centre for Disease Prevention and Control, concluding that the risk was high that the healthcare system’s capacity would be overwhelmed in Europe, recommended teleworking and the closure of schools [10]. On 14 March, 2020, the French Prime Minister announced the closure of all “places receiving public that are not essential to the life of the country” [11]. The activity of primary care (e.g., that performed by independent community midwives and general practitioners, among others) was reduced and urgently reorganised in an attempt to ensure women’s access to care [12, 13]. A subsequent survey showed that more than 90% of independent midwives postponed or cancelled consultations that were deemed non-essential [12]. The pandemic coincided with the entry into effect of the law making the EPI compulsory (1 May 2020) and raised concerns about the disorganisation of care, the problems in referring women who need it, and the loss of opportunity for these future mothers and their children. The French government ended the first lockdown on 11 May, 2020.

The main objectives of this survey were to estimate the proportion of independent midwives who found it harder during lockdown to refer the women in their care to healthcare facilities or other caregivers and to collaborate with hospitals. The secondary objective was to estimate the proportion of independent midwives who felt that at least some of refer the women in their care were experiencing a loss of opportunity, that is, that they were affected by an absence or delay of care that risked to damaged their management. Our hypothesis was that this loss of opportunity reported by independent midwives might vary depending on whether they had a solo or group practice.

## Methods

We conducted a nationwide voluntary open e-survey to recruit a convenience sample of independent midwives working in France during the first lockdown. This quantitative study followed the Checklist for Reporting Results of Internet E-Surveys (CHERRIES) to report our data [14].

### Screening and recruitment

The link to the survey was disseminated by e-mail and Twitter to all the independent midwives who had signed up to receive newsletters from either the French national college of midwives (*Collège National des Sages-Femmes de France*) or the French union of midwives (*Organisation Nationale Syndicale des Sages-Femmes*). A nonprobability chain-referral sampling was obtained by e-mail. All midwives gave their consent before participating in the study, as described in the ethics approval section. Participation was voluntary, without any incentive or reward. The survey was open during the last week of the first lockdown period, from 29 April to 15 May, 2020. Only one entry was accepted from any IP address, to limit the risk of multiple participation.

### Ethics statement

The questionnaire was anonymous. Participants gave their informed consent by participating in the study, which they could stop at any time by leaving the site, thereby withdrawing their permission. The start of the questionnaire clearly stated the objectives of the study, the estimated length of time completing the survey would take, where and for how long data were stored, who the investigators were, the Ethics Committee registration number, and the procedure for submitting objections to such research to the national authorities. This study was approved by the Ethics Committee of the *Hospices Civils de Lyon* (decision n°20–48 dated 23^rd^ March 2020) and registered at the *Comission nationale de l’informatique et des libertés* (CNIL, MR-004 n° 2217640 dated 17 April 2020).

### Survey instrument

We validated the questionnaire tool in four steps. First, the co-authors conceptualised and designed the questionnaire, based on COVID-19 French guidelines for outpatient care and their expertise [15]. Second, it was revised and validated by the Accord group (a multi-professional group whose objective is to Assemble, Coordinate, Understand, Research, and Debate in Primary Care) [16]. Then, it was tested on 10 midwives to verify the items’ usability, technical functionality, clarity, and reliability. It was then administered with open-source software (LimeSurvey). In a last step, we tested the software tool and its functionality.

The survey contained 19 questions on 4 pages. Six focused on the midwives’ characteristics (i.e., age, gender, practice setting: solo or group, and if the latter, whether all the co-workers were also midwives), 2 concerned practice adaptations (cancelled or postponed pregnancy consultations and/or Early Prenatal Interviews), 6 concerned making referrals for women, 4 concerned collaborations with hospitals, and 1 concerned a loss of opportunity for their patients. Loss of opportunity was defined as an absence or delay in care that could damaging their management (definition included in the question).

First, we assessed the difficulties for midwives in making referrals for the women in their care. We estimated these difficulties by using multiple-choice questions on a Likert-like scale (i.e., “no difficulty”, “difficulties, same as before”, and “new difficulties during the pandemic”; see Additional file 1) for each of the six dimensions measured to assess difficulty of access to ambulatory care (i.e., access to psychologists, social workers, family physicians, medical test laboratories, and sonographers).

Then, we assessed the difficulties for midwives in collaborating with hospitals. We estimated these difficulties using multiple-choice questions on a Likert-like scale (i.e., “nonexistent”, “worse than before”, “same as before”, or “better than before”; see Additional file 1) for each of the four dimensions assessed to measure their difficulty of collaborating with the hospital (i.e., transmission of health results, requests for medical expertise, organisation of unscheduled hospital care, and adoption of common protocols).

Finally, we assessed the midwives’ opinions of whether the pandemic had resulted in a “loss of opportunity” for the women in their care, by asking them directly if they considered that “all women (at different levels)” had experienced such a loss, specifically defined in the question as an absence or delay of care that of care that risked damaging their management)”.

All items were mandatory. They were not randomised or alternated. There were no more than 10 items per page to avoid discouraging the respondent and to improve the completion rate of the survey. Respondents were not able to review or change their answers. The questionnaire is available in Additional file 1. We did not use cookies. IP addresses registered in LimeSurvey have not been extracted.

### Statistical analysis

Only completed questionnaires were analysed. All statistical analyses were performed with R software, version 4.2.0 [17]. Depending on their distribution (normal or not), quantitative variables were expressed as means ± standard deviations (SD), and then compared with a Welch two-sample t-test, or as medians [25^th^-75^th^ percentiles] and then compared with a Wilcoxon rank sum test. Qualitative variables were expressed as counts (percentages) and then compared two by two with Fisher’s exact test.

We used binomial logistic regression model to examine factors that predicted loss of opportunity from the midwives’ perspective. All personal (i.e., experience, office practice, and crisis area) and organisational variables were included in the multivariate model except for the gender. All statistical tests were two-sided.

## Results

Complete responses were received from 1491 midwives of the 5264 (28.3%) midwives registered as independent practitioners in France according to the French Midwifery Council. Among the participants, 916 (61.4%) worked in groups, and 159 (11.4%) cancelled or postponed EPI consultations during the lockdown (Table 1).

**Table 1:** Characteristics of respondents compared to all independent midwives registered in France (*n* = 5264)

Characteristics	Survey		Registered^b^		
	*n* = 1491		*n* = 5264		*p*-value
Age in years, median [25-75.^th^ percentile]	41	[34–50]	39	[31–50]	< 0.001
Experience in years, median [25-75.^th^ percentile]	18	[10–27]			
Female gender, n (%)	1463	(98.12)	5064/5204	(97.31)	0.077
Office practice, n (%)					
Single	575	(38.56)			
In midwife-only group	386	(25.89)			
In a multi-disciplinary group	530	(35.55)			
Early-pandemic area, n (%)	609	(40.8)	1762	(33.5)	< 0.001
Cancelled or postponed pregnancy consultation, n (%)	27/1375^a^	(1.96)			
Cancelled or postponed Early Prenatal Interviews, n (%)	159/1394^a^	(11.41)			

^a^Denominators differ because not every midwife practiced all types of consultation before the pandemic

^b^Data kindly provided by the National Chamber of the French Midwifery Council

Overall, 964 independent midwives (64.7%) reported that they had new difficulties in making referrals for women during the pandemic. More specifically 931 (62.4%) had new difficulties in referring them for at least one type of outpatient care, and 33.1% reported difficulty making referrals to social workers (the care providers most frequently reported to be a source of new difficulty). In all, 241 midwives (16.2%) reported new difficulties in referring women to hospital care (Table 2).

**Table 2:** Referral from community midwives (*n* = 1491)

	No difficulty n (line %)	Same difficulty as before n (line %)	New difficulties during the pandemic n (line %)
**Referral to ambulatory care**			
to social workers	580 (38.90)	418 (28.03)	493 (33.07)
to specialist physicians	644 (43.19)	387 (25.96)	460 (30.85)
to psychologists	691 (46.34)	362 (24.28)	438 (29.38)
to medical test laboratories	1149 (77.06)	47 (3.15)	295 (19.79)
to sonographers	1235 (82.83)	105 (7.04)	151 (10.13)
to family physicians	1172 (78.60)	181 (12.14)	138 (9.26)
**Referral to hospital care**	1091 (73.17)	159 (10.67)	241 (16.16)

New difficulties also developed in the collaboration between the hospital and community sectors, reported by 1058 independent midwives (71.0%) concerning at least one of the following dimensions: transmission of health results, requests for medical expertise, organisation of unscheduled care in the hospital, and adoption of common protocols. The greatest deterioration affected the transmission of health data, reported by 26.8% of midwives (Table 3).

**Table 3:** Collaboration quality between hospital and community sectors (*n* = 1491)

n (line %)	None	Worse than before	Same as before	Better than before
Transmission of health data	155 (10.40)	399 (26.76)	886 (59.42)	51 (3.42)
Requests for medical expertise	130 (8.72)	326 (21.86)	913 (61.23)	122 (8.18)
Organisation of unscheduled care in hospital	218 (14.62)	386 (25.89)	820 (55.00)	67 (4.49)
Implementation of common protocols	475 (31.86)	210 (14.08)	677 (45.41)	129 (8.65)

Among the independent midwives who responded to this survey, 689 (46.2%) considered that all the women under their care had experienced a loss of opportunity, to various degrees. These midwives were also those who had the least experience (Table 4). The proportion of midwives considering that women had had a loss of opportunity was similar among those practising alone (270/575, 47.0%) or in a midwife-only group (181/386, 46.9%), and slightly but not significantly lower among those practising in multi-disciplinary groups (238/540, 44.9%; Table 4). This proportion was, however, significantly higher among the midwives who reported having new difficulties in making referrals for their patients to a social worker, to a specialist physician, to the hospital or in obtaining medical expertise from hospital professionals (Table 4).

**Table 4:** Consideration of loss of opportunity for independent midwives by their characteristics or other perceptions

Independent midwives' individual characteristics		No loss of opportunity n, (line %) *N* = 802	Loss of opportunity n, (line %) *N* = 689	OR univariate (95%CI)	OR multivariate (95%CI)^a^
Age	< 40 years	325 (49.8)	328 (50.2)		
	≥ 40 years	477 (56.9)	361 (43.1)	**0.75 (0.61 – 0.92)**	
Experience	< 20 years	429 (51.2)	409 (48.8)		
	≥ 20 years	373 (57.1)	280 (42.9)	**0.79 (0.64 – 0.97)**	**0.78 (0.63 – 0.96)**
Office practice	Single	305 (53.0)	270 (47.0)	1.05 (0.85 – 1.29)	0.69 (0.32 – 1.47)
	In midwife-only group	205 (53.1)	181 (46.9)	1.04 (0.82 – 1.31)	0.98 (0.76 – 1.27)
	In a multi-disciplinary group	292 (55.1)	238 (44.9)	0.92 (0.74 – 1.14)	0.87 (0.69 – 1.11)
Crisis area	Early pandemic area	328 (53.9)	281 (46.1)		
	Later pandemic area	474 (53.7)	408 (46.3)	1.00 (0.81 – 1.22)	1.00 (0.81 – 1.23)
**Independent midwives' organisational characteristics**					
Adaptation of obstetric consultations	Maintained	728 (53.8)	620 (46.2)		
	Postponed	15 (55.6)	12 (44.4)	0.93 (0.42 – 2.00)	
Adaptation of Early Prenatal Interviews	Maintained	715 (53.7)	617 (46.3)		
	Postponed	87 (54.7)	72 (45.3)	0.96 (0.69 – 1.33)	
Referral to social worker	None or same	558 (55.9)	440 (44.1)		
	New difficulties	244 (49.5)	249 (50.5)	**1.29 (1.04 – 1.61)**	
Referral to specialist physician	None or same	577 (56.0)	454 (44.0)		
	New difficulties	225 (48.9)	235 (51.1)	**1.33 (1.07 – 1.66)**	
Referral to psychologist	None or same	573 (54.4)	480 (45.6)		
	New difficulties	229 (52.3)	209 (47.7)	1.09 (0.87 – 1.36)	
Referral to medical test laboratory	None or same	658 (55.0)	538 (45.0)		
	New difficulties	144 (48.8)	151 (51.2)	1.28 (0.99 – 1.66)	
Referral to sonographer	None or same	731 (54.6)	609 (45.4)		
	New difficulties	71 (47.0)	80 (53.0)	1.35 (0.97 – 1.90)	
Referral to family physician	None or same	738 (54.5)	615 (45.5)		
	New difficulties	64 (46.4)	74 (53.6)	1.39 (0.98 – 1.98)	
Referral to ambulatory care	None or same	319 (57.0)	241 (43.0)		
	New difficulties	483 (51.9)	448 (48.1)	1.23 (0.99 – 1.52)	
Referral to hospital care	None or same	693 (55.4)	557 (44.6)		
	New difficulties	109 (45.2)	132 (54.8)	1.17 (0.94 – 1.47)	
Transmission of health results	None or same	598 (54.8)	494 (45.2)		
	New difficulties	204 (51.1)	195 (48.9)	1.16 (0.92 – 1.46)	
Requests for medical expertise	None or same	646 (55.5)	519 (44.5)		
	New difficulties	156 (47.9)	170 (52.1)	**1.36 (1.06 – 1.74)**	
Organisation of unscheduled care in hospital	None or same	606 (54.8)	499 (45.2)		
	New difficulties	196 (50.8)	190 (49.2)	1.18 (0.93 – 1.48)	
Adoption of common protocols	None or same	682 (53.2)	599 (46.8)		
	New difficulties	120 (57.1)	90 (42.9)	0.85 (0.63 – 1.15)	

^a^multivariate analysis including experience, office practice, and crisis area

Contrary to our initial hypothesis, the logistic regression does not reveal any significant variation in the evidence of loss of opportunity reported by independent midwives depending on whether they had a solo or group practice. The only individual characteristic of the midwives that was found to be associated with this perceived loss of opportunity was the experience of the caregivers. The most experienced midwives were the least inclined to report a risk of loss of opportunity for the women they cared for (Table 4).

## Discussion

### Main findings

This study shows that two-thirds of independent midwives who responded to the questionnaire reported that it was harder than before the pandemic to make referrals of women to other healthcare workers and/or to collaborate with the hospital. Almost half felt that all of the women they cared for could have been harmed (to different degrees) by of the absence or delay of care. This proportion was even higher among those with a lower level of experience and those who reported difficulties in making referrals to social workers, specialist physicians, or to hospitals, but did not differ according to whether they practised solo or in a group.

### Strengths and limitations

The number of participants exceeds the number of midwives who received the survey by newsletters. This result demonstrates its wide dissemination, well beyond this initial distribution channel. This is one of this study’s strengths. Another is that the midwives were questioned during the last week of the lockdown, thereby preventing a risk of memory bias.

One of the study’s main limitations is probably the selection bias, inherent in internet surveys and the similarly inherent social desirability bias. The midwives practicing in the areas most affected by the pandemic responded most often. However, given that senior midwives often respond less to such online surveys, we were surprised to observe that the median age of respondents was slightly higher than the average age of registered midwives. This is possibly due to the fact that younger midwives were more likely to be busy both working and caring for their children, as schools were closed. Nonetheless, these biases did not prevent the emergence of some valuable findings, as discussed in the interpretation.

### Interpretation

A few key findings warrant highlighting. First, although nearly 90% of the respondents continued their preventive consultations (specifically, the Early Prenatal Interviews), most reported new difficulties in making referrals to at least one type of outpatient care during the COVID-19 lockdown. This raises questions about collaboration between primary and secondary care actors, and it is particularly worrying because the midwives who reported new difficulties were also those who most often reported a perceived loss of opportunity for the women under their care. Second, solo practice did not appear to be strongly related to these coordination difficulties and their impact on women’s loss of opportunity.

First, our results raise questions about collaboration between primary and secondary care actors, especially during an ongoing crisis. The COVID-19 pandemic has revealed the unpreparedness of healthcare delivery systems around the world [18]. In France in 2020, 41% of midwives were community midwives (i.e., primary care), while 59% worked in hospitals (i.e., primary and secondary care) [19]. Births in France take place almost exclusively in hospitals (99.4%), but antenatal care (pregnancy follow-up), which is generally considered primary care, takes place mainly outside hospitals (69.2%) [5, 20]. The first public health measures taken by the French public authorities in relation to the pandemic did not involve the community primary care sector (i.e. independent midwives, private gynaecologist-obstetricians, or family physicians) responsible for this antenatal care [16]. The same exclusion has been described in Australia, where midwives and family physicians are also independent healthcare providers [21, 22]. Our study shows that the midwives who reported a deterioration in collaboration between primary and secondary care, i.e., a deterioration in the care pathway for pregnant women, are also those who considered that the women under their care were likely to have experienced a loss of opportunity for their health. These results are consistent with the literature, which shows an increase in women’s isolation and psychological difficulties during the pandemic [23–25]. Beyond women’s health, the health of children, particularly in relation to the morbidity associated with prematurity, is also likely to be improved by supporting mothers at psychosocial risk [26]. This is even more regrettable because the literature has shown that the quality of care can be improved by ensuring that primary and secondary care are well linked [27].

One might think that these collaboration difficulties are due to the isolated solo practices of independent community healthcare workers. However, in France, interestingly, salaried community midwives and social workers are employees of public structures and work within them. But while almost all independent community midwives maintained their activity during the lockdown, salaried community midwives and social workers were obliged by their employers to stop working for several weeks in order to reorganise before resuming their activity [28]. The independent system appears to be more flexible and responsive than the state model.

The creation of perinatal networks more than a decade ago in France has substantially improved practices and collaboration by enabled hospital caregivers from different professions (midwives, obstetricians, paediatricians, anaesthesiologists) and different institutions to work together (e.g. to adopt common care protocols). Providers in private, independent practices (e.g. independent midwives, private gynaecologist-obstetricians, family physicians, …) remain largely excluded from these networks, although the results of our study suggest that their inclusion would raise the quality of care still farther. The compartmentalisation of the French primary care and hospital sectors is therefore questionable.

An important research perspective of this study on the interface between primary and secondary care countries such as France, the main causes of maternal death are now cardiovascular disease and suicide rather than haemorrhage [29–33]. Most of the suicide deaths occurred mainly in the postpartum period, and many of both these causes of death were attributable to lack of connection and communication between the primary and hospital health care providers. The screening, diagnosis, and care of both cardiovascular and psychiatric diseases take place mainly in the community, even during pregnancy. As the CNEMM strongly recommended, the relations between community care and hospitals must change in these cases to save lives [34, 35]. Health services research, and especially primary care transition research, should be promoted as an opportunity to improve the health of populations and more specifically of mothers [36].

## Conclusions

Consultation referrals and collaboration have been affected by the lockdown, with a perceived impact on the health of pregnant women. Independent midwives should be better integrated into the perinatal health system and the perinatal networks.

## Supplementary Information

**Additional file 1.** Auto questionnaire.

**Additional file 2. **

## Data Availability

The data that support the findings of this study are available in supplementary material.
