# Supplementary material for: The challenge of care coordination by midwives during the COVID-19 pandemic: a national descriptive survey
Source: BMC Pregnancy Childbirth. 2022 May 25;22:437. doi: 10.1186/s12884-022-04772-2 (PMC9131711; doi:10.1186/s12884-022-04772-2)
Supplement: Supplementary file 1 — Additional file 1. Auto questionnaire. [file 12884_2022_4772_MOESM1_ESM.docx]

**Appendix: Auto questionnaire**

**Section1: characteristics**

1. How old are you?
2. How many years of professional experience do you have?
3. Are you

- Male
- Female

1. What district do you practice in?
2. Is your practice …

- Your own solo practice?
- A group practice?

1. Specify the nature of your group practice

- All midwives
- Multiple medical/paramedical professions

**Section 2: Adaptation of practices**

1. Have you cancelled or postponed pregnancy follow-up consultations?

- Yes
- No

1. Have you cancelled or postponed Early Prenatal Interviews?

- Yes
- No

**Section 3: Referral to care**

1. Have you had trouble in referring women to a psychologist?

- No difficulties
- New difficulties, during the pandemic
- Difficulties, same as before

1. Have you had trouble in referring women to a social worker?

- No difficulties
- New difficulties, during the pandemic
- Difficulties, same as before

1. Have you had trouble in referring women to a specialist physician?

- No difficulties
- New difficulties, during the pandemic
- Difficulties, same as before

1. Have you had trouble in referring women to medical test laboratories?

- No difficulties
- New difficulties, during the pandemic
- Difficulties, same as before

1. Have you had trouble in referring women to a sonographer?

- No difficulties
- New difficulties during the pandemic
- Difficulties, same as before

1. Have you had trouble in referring women to the hospital?

- No difficulties
- New difficulties, during the pandemic
- Difficulties, same as before

**Section 3: Collaboration with hospital**

1. How would you evaluate the transmission of health results (hospital report, ultrasound, laboratory, etc.)?

- None
- Worse than before
- Same as before
- Better than before

1. How would you evaluate the communication between caregivers (request for medical expertise)?

- None
- Worse than before
- Same as before
- Better than before

1. How would you evaluate the organisation of unscheduled hospital care?

- None
- Worse than as before
- Same as before
- Better than before

1. How would you evaluate the adoption of common protocols?

- None
- Worse than before
- Same as before
- Better than before

**Section 4: Loss of chance**

1. Do you think that all women (at different levels) had a loss of opportunity during the crisis (absence or delay of care that risked damaging their management)?

- Yes
- No
